# Influence of food commodities on hangover based on alcohol dehydrogenase and aldehyde dehydrogenase activities

**DOI:** 10.1016/j.crfs.2019.09.001

**Published:** 2019-09-17

**Authors:** Shraddha Srinivasan, Kriti Kumari Dubey, Rekha S. Singhal

**Affiliations:** Food Engineering and Technology Department, Institute of Chemical Technology, Matunga, Mumbai 400019, India

**Keywords:** Fruit juice blends, Alcohol dehydrogenase (ADH), Aldehyde dehydrogenase (ALDH), Anti-hangover formulation, Kinetics

## Abstract

Alcohol consumption often leads to hangover, a condition characterized by several symptoms, characteristically headache, nausea, fatigue and drowsiness. Hangover may be alleviated by altering the rate of alcohol metabolism and facilitating elimination of acetaldehyde by affecting the activity of alcohol dehydrogenase (ADH) and/or aldehyde dehydrogenase (ALDH) enzymes. In the present study, several food commodities like fruits, vegetables, cereals, pulses, dairy products, spices and other miscellaneous products (ascorbic acid, cocoa sample, tea, coffee, egg yolk and date samples) were investigated for their effect on the *in vitro* activities of the enzymes and their antioxidant properties. Of the many screened food commodities, few showed an increase in the activity of either one or both the enzymes, ADH and ALDH. Studies showed no correlation between ADH and ALDH enzyme activities and antioxidant property of the selected food commodities for anti-hangover effect. Further, an anti-hangover (AHO) product was developed using pear (65%), sweet lime (25%) and coconut water (10%) and, validated for *in vitro* ADH and ALDH enzyme activities. AHO product was found to enhance ADH and ALDH activities by 23.31% and 70.02%, respectively.

## Highlights

• Screening of food influencing alcohol dehydrogenase (ADH) and aldehyde dehydrogenase (ALDH) activities.
• Foods commodities enhancing ADH and/or ALDH activities can be used in AHO formulation.
• Pear (65%), sweetlime (25%) and coconut water(10%) used in anti-hangover (AHO) formulation.
• AHO product had increased ADH (23.3%) and ALDH (70.02%) activities.

## Graphical abstract

**Figure:**
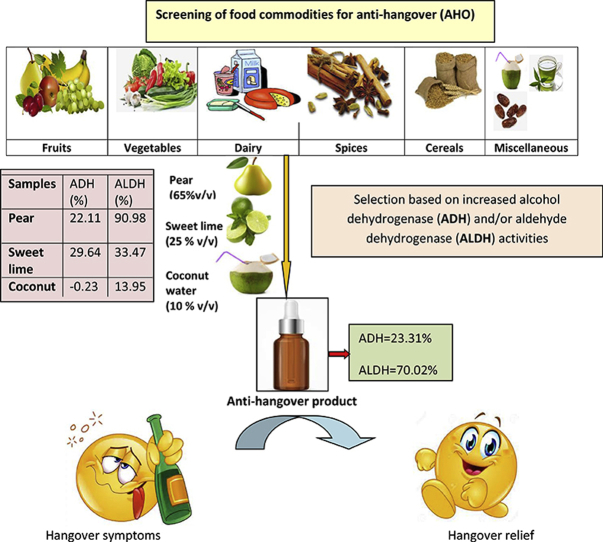

## 1 Introduction

Hangover or veisalgia is the term that refers to the psychological and physiological effects following alcohol consumption. It is characterized by an unpleasant and uneasy feeling that includes, but is not limited to, headache, fatigue, drowsiness, nausea, and in some cases, vomiting (Swift and Davidson, 1998). The symptoms set in when the concentration of ethanol in the blood reaches zero, implying that it is the metabolism or the metabolite that leads to this effect. The effects of hangover are attributed to several causes, the major ones being due to i) the direct effects of alcohol, ii) metabolism of alcohol, iii) other non-alcohol factors such as the presence of congeners (biologically active molecules present in alcohol, apart from ethanol, for example, methanol or ethyl formate), and iv) genetic makeup. Ethanol consumption affects specific organs or systems such as the brain, gastrointestinal tract, liver and immune system (Swift and Davidson, 1998) manifesting as symptoms that are characteristic of a typical hangover.

During metabolism of ethanol, it gets broken down either through the oxidative or non-oxidative pathway (Zakhari, 2006). During oxidative metabolism of alcohol to acetate, ethanol is converted to acetaldehyde by the hepatic alcohol dehydrogenase (ADH). Nicotinamide adenine dinucleotide (NAD^+^) serves as the intermediate electron carrier for ADH, and it gets reduced to nicotinamide adenine dinucleotide-reduced (NADH). The increase in the NADH causes oxidative stress in the liver which also contributes to the symptoms exhibited during the phase of hangover. Acetaldehyde built-up has been attributed as major causative factor of hangover (Zakhari, 2006). The alleles of the ADH and ALDH form the genetic basis for the manifestation of hangover. The genetic effect is predominantly seen among the racial populations (Cederbaum, 2012, Quertemont, 2004). The isozymes of ADH and ALDH have different affinity for their substrates, and thus the genetic makeup plays a major role in determining the rate at which an individual will eliminate ethanol from the system.

Hangover poses a considerable threat at the individual level, where along with the occurrence of unpleasantness there is also a risk of health issues that might impact everyday functioning. It also affects the economy due to a decrease in the productive work caused by sleep deprivation, loss of attentiveness and alertness (Cederbaum, 2012). Several natural compounds like 6-gingerol (Takahashi et al., 2010), dehydroevodiamine (Wang et al., 2016a), ginsenosides (Kim et al., 2011), linolenic acids (Lee et al., 2014a) among many others, have been recommended as a cure for hangover. Researchers have investigated and identified natural components (such as polyphenols) from various food sources such as fruits, mushroom, and herbs to alleviate hangover symptoms (Zhao et al., 2017, Zhang et al., 2016, Kim, 2016, Bajpai et al., 2016). These natural food sources are reported to function effectively by exhibiting positive effects on the hepatic enzymes, i.e. by either enhancing the activity of ALDH and/or ADH and thereby assisting in aldehyde and alcohol clearance from the system, respectively. A herbal mixture “DTS 20” containing *Viscum album* L. (40%), *Lycium chinense* L. (30%), *Inonotus obliquus* (20%), and *Acanthopanax senticosus* H. (10%) has also been studied and found to reduce the oxidative stress and plasma alcohol concentrations (Hong, 2015).

While pharmaceutics have also gained momentum in the search to find a cure for hangover, the identification of natural compounds will target the implementation of dietary modifications to ensure better results. This research work aims at studying the influence of food commodities on ADH and ALDH enzyme activities and their kinetic properties that could further help to identify specific components to formulate an effective natural AHO product. Few studies have observed that sugars like glucose and fructose do alleviate hangover by facilitating alcohol detoxification. However, these do not result through the action of the hepatic enzymes (ADH and ALDH) and are a separate entity for research scope.

## 2 Materials and methods

### 2.1 Sample procurement

The fruits, vegetables, cereals, pulses, dairy products, spices and other miscellaneous products were purchased from the local market of Mumbai city, India. The food commodities used are listed in Table 1.

**Table 1:** List of food commodities screened for AHO formulation.

Fruits
Carambola/starfruit (*Averrhoacarambola*), orange (*Citrussinensis* L.), gooseberry *(Phyllanthus emblica*), black grapes *(Vitis amurensis*), green grapes *(Vitis vinifera*), pitahaya/dragonfruit *(Hylocereus undatus)*, pear *(Pyruscommunis)*, Apple *(Malus pumila*), sweet lime *(Citrus limetta*), mango *(Mangifera indica*), kiwifruit *(Actinidia deliciosa*), pineapple *(Ananas comosus*), papaya *(Carica papaya*), lemon (*Citrus limon* L.), watermelon (*Citrullus lanatus*), pomegranate (*Punica granatum)*
Vegetables
Garlic (*Allium sativum*), cucumber (*Cucumis sativus*), coriander (*Coriandrum sativum*), fenugreek leaves (*Trigonella foenum-graecum*), spinach (*Spinacia oleracea*), bitter gourd/bitter melon (*Momordica charantia*), carrot (*Daucus carota*), Onion (*Allium cepa*), tomato (*Solanum lycopersicum*)
Cereals
Sorghum (*Sorghum* sp. bicolor), oats (*Avena sativa*), wheat (*Triticum aestivum*), maize (*Zea mays*), peanuts (*Arachis hypogaea*)*,* millet (*Pennisetum glaucum*)
Spices
Black pepper, cassia, cinnamon, cloves, ginger, cumin (jeera), mace, nutmeg, turmeric
Milk Products
Butter-milk, cheese, milk, a commercial probiotic drink
Miscellaneous
Ascorbic acid, black tea, cocoa, coconut water, coffee, dates, egg white egg yolk, fenugreek seeds, green tea

### 2.2 Chemicals and reagents

2,2 Diphenyl-1-picrylhydrazyl (DPPH), ADH and ALDH were procured from Sigma-Aldrich, Bangalore, India. Bovine serum albumin (BSA), β-mercaptoethanol, potassium chloride, gallic acid, β-nicotinamide adenine dinucleotide (NAD^+^) and NADH were purchased from HiMedia Laboratories, Mumbai, India. Ethanol, methanol, and acetaldehyde were purchased from Merck Ltd, Mumbai, India.

### 2.3 Preparation of samples

#### 2.3.1 Fruits, vegetables, spices and cereals

The fruits and vegetables were washed, cleaned and dried. The fruit/vegetable juices were extracted by grinding/pulping them in a mixer/grinder and filtered through Whatman filter No. 2 as samples for further experiments. Spices were ground to size ≤0.25 mm and extracted in a 1% (w/v) solution of 60% methanol using magnetic stirrer for 1 h. The extracts were filtered through Whatman No. 1 filter paper, dried and resuspended in deionized water at a concentration of 100 mg/mL (Subbaraj et al., 2016). Cereals and pulses samples were prepared by following the method as per Meneses et al. (2013) with some modifications. Cereals and pulses were milled to a particle size of ≤0.25 mm and stored in refrigerator prior to sample preparation. The sample was prepared by taking 10% w/v solution of the cereals or pulses in 60% methanol and subjected to magnetic agitation for 30 min at 60 °C. The obtained extracts were then filtered, dried and resuspended in a similar fashion as the spice samples. All the fruits, vegetables, spices and cereal samples were stored at −20 °C and used for various assays within two days. Fruits and vegetables were used directly as juices since the constituents are generally homogenized and most of the bioactive compounds are available for the reaction. However, for spices the components need to be extracted using suitable solvents so as to make them available for the reaction. Similarly, dairy products were diluted in order to prevent interference from particulate matter. In the current study, extraction of the components that would also exhibit antioxidant properties were aimed at so as to test for their effect on the hepatic enzymes and also to check for their antioxidant activity.

#### 2.3.2 Dairy products

Extracts of milk and cheddar cheese were obtained by following the protocol as per Pritchard (Pritchard et al., 2010) with modifications. Briefly, Cheddar cheese was homogenized in a ratio of 1:3 w/v with deionized water and was subjected to magnetic agitation at 40 °C for 1 h. The contents were then centrifuged at 4250 g at 4 °C for 30 min. The supernatant was filtered through Whatman filter paper (No. 42) followed by membrane filtration (pore size 0.22 μm). Diluted samples (1:1 v/v) of buttermilk and commercial probiotic product were used after filtration through a membrane of pore size 0.45 μm. Samples were stored at −20 °C in deep freezer and used for assays within two days.

#### 2.3.3 Miscellaneous

Ascorbic acid was used at the concentration of 2 mg/mL for various assays. Cocoa sample (1:100) was prepared in 80% methanol solution with stirring (1 h) followed by solvent evaporation and resuspension in deionized water. Tea and coffee (0.1 g) were brewed with 8 mL boiling deionized water for 2 min (Floegel et al., 2011) and filtered through Whatman filter paper and membrane filtration (0.45 μm membrane). For egg yolk, sample preparation was done as per the method reported by Su et al. (2015). Egg yolk was separated from egg white and beaten with addition of 95% ethanol in 1:10 ratio. The contents were then mixed in a magnetic stirrer at 65 °C for 1 h and filtered, the process was repeated twice. The ethanol fractions were pooled and ethanol was evaporated. Deionized water was added to the residue and the sample was assayed within two days. Date sample was prepared by measuring 1 g of seedless dates and mixing it in 10 mL of deionized water for 48 h under stirring conditions. The contents were then mixed in a mixer and centrifuged at 4000 rpm (Remi Centrifuge, REMI- CRP-30, Mumbai, India) at 4 °C for 20 min. The supernatant was collected further and used for assays (Zangiabadi et al., 2011). All the processed samples were stored at −20 °C until the assays were performed.

### 2.4 Enzymatic assays for screening of food commodities

#### 2.4.1 Determination of ADH activity

The ADH activity was determined according to the protocol reported by Lee et al. (2012) with slight modifications. The following solutions were prepared: 1 M Tris HCl (adjusted to pH 7.5 with 1M HCl at 25 °C), freshly prepared 3 mM β NAD^+^, 12.5 mM ethanol solution, 0.5% Triton X 100, 100 mM Tris HCl buffer with 0.02% (w/v) BSA at pH 8.0, 20 U/mL ADH (prepared in buffer) and 1 mL of the test sample extract. The *in vitro* activity of ADH was measured as NADH formed, determined spectrophotometrically at 340 nm using an ELISA plate reader (BioTek ELx808, Mumbai, India). The reaction was started by addition of ethanol to a pre-incubated reaction mixture containing the remaining solutions and made-up to a final volume of 300 μL. The reaction was carried out at 25 °C and pH of 7.5. The control reaction was carried out in the absence of any test sample. A blank reaction (in the absence of the substrate) was also measured and values were corrected with respect to the baseline in order to eliminate the effect of any artefacts.

#### 2.4.2 Determination of ALDH activity

The ALDH activity was also determined according to the protocol mentioned by Lee et al. (2012) with slight modifications. The following solutions were prepared: 1 M Tris HCl (adjusted to pH 8.0 with 1M HCl at 25 °C), freshly prepared 3 mM β NAD+, 100 mM acetaldehyde solution, 3 M KCl, freshly prepared 1 M β-mercaptoethanol solution, 100 mM Tris HCl buffer with 0.02% (w/v) BSA at pH 8.0, 0.1 U/mL ALDH (prepared in buffer) and 1 mL of the test sample extract. The *in vitro* activity of ALDH was measured as NADH formed, determined spectrophotometrically at 340 nm using an ELISA plate reader (BioTek ELx808, Mumbai, India). The reaction was started by addition of acetaldehyde to a reaction mixture of the other solutions and made-up to a final volume of 300 μL. The reaction was carried out at 25 °C and pH of 8.0. The control reaction was carried out in the absence of any test sample. A blank reaction (in the absence of the substrate) was also measured and values were corrected with respect to the baseline in order to eliminate the effect of any artefacts. The percent activity of ADH and ALDH was calculated by the formula.

$$Percent\;enzyme\;activity=\frac{\left(NADH\;formed\;in\;sample\;reaction-NADH\;formed\;in\;control\;reaction\right)}{NADH\;formed\;in\;sample\;reaction}$$

### 2.5 Antioxidant potential

Antioxidant property of a substance was assayed by the 2,2-Diphenyl-1-picrylhydrazyl radical (DPPH•) scavenging assay (Ramadan et al., 2008) using a spectrophotometer at 517 nm. Gallic acid was used as a standard. The percentage inhibition was calculated using the formula given.

$$Percent\;radical\;scavenging\;activity=\frac{\left(OD\;of\;control-OD\;of\;sample\right)}{OD\;of\;control}$$

The antioxidant capacities for the samples were reported as gallic acid equivalent (GAE).

### 2.6 Kinetic studies

The activation kinetics of the samples that positively affected the activity of ADH and ALDH were studied and the apparent K_m_ value was calculated.

### 2.7 Correlation between antioxidant property and enzyme activity

The correlation between ADH activity, ALDH activity and antioxidant property of the samples were determined by plotting a scatter plot and calculating the R^2^ values.

## 3 Theory/calculation

An AHO product was developed based on the effect of the substances on ADH activity, ALDH activity and organoleptic acceptability. D-optimal mixture design was applied to find the optimal response for any mixture of the selected substances, and to obtain the influence on the response of the combination of substances. Percentage of components; pear (A), sweet lime (B) and coconut water (C) were the three independent parameters. The dependent variables selected as the response was overall average acceptability score given by the sensory panel comprising of 15 panellists (8 females and 7 males in the age group of 22–28 years). The scoring was based on 9-point hedonic scale as follows: 9-like extremely; 8-like very much; 7-like moderately; 6-like slightly; 5-neither like nor dislike; 4-dislike slightly; 3-dislike moderately; 2-dislike very much; 1-dislike extremely.

The statistical software package Design Expert 7.0 (Stat-Ease Inc., Minneapolis, MN, USA), was used for the experimental design and data analysis. All the analyses were carried out in triplicates and the data generated has been reported in terms of mean ± standard deviation (SD). Data were assessed by ANOVA (Analysis of Variance) to determine the significance of difference observed in the samples. The significance for the mean difference for all the data generated and reported was compared using Duncan's multiple comparison test (p < 0.05).

## 4 Results and discussion

Food samples necessarily enhancing the ADH and/or ALDH are suitable for AHO formulation.

### 4.1 Effect of fruits and vegetables on alcohol dehydrogenase (ADH) activity and aldehyde dehydrogenase (ALDH) activity

Fruits and vegetables that decrease the ALDH activity are not suitable as an AHO substance. The effect of fruits and vegetables on the percent difference in activities of ADH and ALDH is shown in Table 2. Grapes, dragon fruit and gooseberry did not significantly affect the activity of ADH while starfruit and orange significantly decreased the activity of ADH. In the present work, the high percentage activity difference between the watermelon sample reaction and control reaction indicates a positive effect of watermelon in enhancing the activity of ADH by 67.22%. Grapes did not alter the ALDH activity, while gooseberry decreased it. Studies performed by Zhang et al. (2016) also confirmed the ability of starfruit to decrease the ADH activity, though not very significantly. A commercially available AHO product “PartySmart” containing grapes and gooseberry as ingredients has been reported to have a positive effect on both ADH and ALDH (Pordié, 2015). Wang et al. (2016b) observed fresh orange to decrease the ADH activity. Similarly, mango pulp has also been reported to enhance the ADH activity in rats by 129.76% (Kim et al., 2011). However, Zhang et al. (2016) did not find any significant difference in the ADH activity by watermelon and lemon, *Actinidia chinensis* (closely related to kiwi fruit). While the difference in the reported results from that of the present study can be attributed to variations in the samples used, it could also be that *in vitro* and *in vivo* studies have yielded different results.

**Table 2:** Effect of fruits and vegetables on ADH and ALDH activities.

Food commodities	ADH activity (%)		ALDH activity (%)
Fruits			
*Malus pumila* (apple)	28.06 ± 0.95^i^	−76.15 ± 1.46^b^	
*Mangifera indica* (mango)	31.74 ± 1.65^i^	−61.97 ± 1.08^c^	
*Citrullus lanatus* (watermelon)	67.22 ± 2.81^m^	−57.39 ± 1.38^c^	
*Carica papaya* (papaya)	46.50 ± 3.73^k^	−50.55 ± 2.24^d^	
*Vitis amurensis* (black grapes)	−1.36 ± 0.26^de^	−33.44 ± 2.03^fg^	
*Phyllanthus emblica* (gooseberry)	−2.73 ± 0.22^d^	−31.42 ± 2.20^g^	
*Ananas comosus* (pineapple)	39.28 ± 2.98^j^	−30.35 ± 3.06^g^	
*Punica granatum* (pomegranate)	67.97 ± 6.95^m^	−22.48 ± 4.75^h^	
*Actinidia deliciosa* (kiwi fruit)	38.80 ± 6.29^j^	−20.09 ± 4.48^h^	
*Citrus limon (L.)* (lemon)	47.39 ± 8.76^k^	−19.59 ± 4.12^h^	
*Hylocereus undatus* (dragon fruit)	4.57 ± 0.38^e^	−3.06 ± 1.44^j^	
*Vitis vinifera* (green grapes)	3.02 ± 0.16^def^	−0.20 ± 0.02^j^	
*Citrus sinensis (L.)* (orange)	−8.54 ± 1.91^c^	15.48 ± 5.16^l^	
*Averrhoa carambola* (starfruit)	−13.39 ± 1.18^ab^	22.76 ± 9.26^i^	
*Citrus limetta* (sweet lime)	29.64 ± 7.36^i^	33.47 ± 1.38^n^	
*Pyrus* sp. (pear)	22.11 ± 3.21^h^	90.98 ± 1.96^p^	
Vegetables			
*Allium sativum* (garlic)	−44.11 ± 4.32^a^	−42.23 ± 2.72^e^	
*Cucumis sativus* (cucumber)	−18.37 ± 2.26^b^	87.25 ± 1.55^p^	
*Coriandrum sativum* (coriander)	−12.36 ± 0.70^c^	−58.38 ± 12.20^b^	
*Trigonella foenum-graecum* (fenugreek leaves)	2.62 ± 0.58^def^	−6.43 ± 1.53^ij^	
*Spinacia oleracea* (spinach)	6.10 ± 0.72f	−38.94 ± 2.27ef	
*Momordica charantia* (bitter gourd/bitter melon)	14.42 ± 1.87^g^	−1.59 ± 0.16^j^	
*Daucus carota* (carrot)	29.33 ± 2.45^i^	−10.76 ± 3.55^i^	
*Allium cepa* (Onion)	48.25 ± 3.51^k^	2.24 ± 0.90^k^	
*Solanum lycopersicum* (tomato)	57.25 ± 2.79^l^	41.19 ± 6.37°	
Control	0.00^def^	0.00 ± 0.00^jk^	
Commercial anti-hangover product	−11.64 ± 1.49^c^	−82.50 ± 0.57^a^	

Values are mean ± SD of three determinants.

Different alphabet in superscript represent that the values are significantly different (p < 0.05).

Pear showed the highest positive effect on ALDH activity at 90.98%. Orange, starfruit, and sweet lime also enhanced the ALDH activity significantly by 15.48%, 22.76% and 33.47%, respectively. The potential use of pear to alleviate hangover has been reported in previous studies based on their effect on the activity of the enzyme (Lee et al., 2012). Enhanced ALDH activity of pear facilitates in faster elimination of acetaldehyde and thus it serves as a suitable AHO. Zhang et al. (2016) reported that orange and starfruit showed a decrease in the activity of ALDH (by 11.81% and 61.95%, respectively) while dragon fruit did not significantly affect the ALDH activity. Wang et al. (2016b) also reported fresh orange juice to decrease the ALDH activity. This variation could be due to the differences in the sample nature and sample preparation methods. The presence of polyphenols in the fruits has been reported to enhance the activity of ADH and ALDH (Lee et al., 2012). However, the exact mechanism is yet to be elucidated.

Among the vegetable samples analysed, bitter gourd and carrot showed increase in ADH activities and decrease in ALDH activities. Tomato and cucumber samples showed an increase in the activity of ALDH (41.19% and 87.25%). Investigators have reported that heat treated cucumber had the ability to enhance the activity of ALDH and ADH (Bajpai et al., 2016). Contrary to this result, our work showed a significant decrease of ADH activity by cucumber. This could be due to differences in the variety as well sample preparation methods.

### 4.2 Effect of dairy products, cereals, pulses, spices and other miscellaneous food products on ADH and ALDH activity

The effect of dairy products, pulses, spices, and other miscellaneous food products on ADH and ALDH activities are shown in Table 3. Dairy products like milk showed a marginal but significant decrease in the activity of ALDH, while the other samples such as buttermilk, cheese and a commercial prebiotic drink showed an increase in the activity of the ALDH. In addition to the ability to enhance the activity of the enzyme, this effect could be attributed to the presence of microorganisms in the samples that are able to produce ALDH and thereby enhance the rate of product formation. *Lactobacillus* sp. fermented cream cheese has been reported as an AHO product due to its ability to increase the rate of acetaldehyde metabolism (Konkit et al., 2016). Moreover, the presence of several functional peptides in these fermented products may contribute to the modified effect on the enzymes.

**Table 3:** Effect of dairy products, cereals, pulses, spices and other miscellaneous food products on ADH and ALDH activity.

Food commodities	ADH activity (%)	ALDH activity (%)
Dairy products		
Milk	−10.75 ± 2.04^i^	−3.93 ± 0.55°
Buttermilk	−4.01 ± 2.15^j^	17.50 ± 0.97^r^
A commercial probiotic drink	−4.04 ± 1.96^j^	24.23 ± 3.14^s^
Cheddar cheese	97.31 ± 6.25^p^	18.63 ± 2.34^r^
Cereals and pulses		
*Avena sativa* (oats)	−24.83 ± 1.53^de^	−76.81 ± 1.15^d^
*Arachis hypogaea* (peanuts)	−15.06 ± 1.44^gh^	−66.41 ± 3.78^e^
*Pennisetum glaucum* (millet)	−1.74 ± 0.58^jk^	−47.13 ± 3.50^h^
*Sorghum* sp. (sorghum)	−26.70 ± 4.24^d^	−32.16 ± 3.11^jk^
*Zea mays* (maize)	−14.38 ± 2.01^ghi^	−0.51 ± 0.13^op^
*Triticum* sp. (wheat)	−17.71 ± 1.13^fg^	0.32 ± 0.21^opq^
Spices		
Pepper	−67.41 ± 2.95^b^	−34.08 ± 1.67^ijk^
Cloves	−67.15 ± 2.06^b^	−60.81 ± 5.58^f^
Nutmeg	−46.21 ± 2.87^c^	−97.89 ± 7.81^a^
Cumin	−23.56 ± 0.80^de^	−3.54 ± 2.23°
Cinnamon	−21.38 ± 1.88^ef^	−61.16 ± 2.02^f^
Cassia	−18.13 ± 2.01^fg^	−21.06 ± 4.61^n^
Mace	13.26 ± 1.67^m^	−82.48 ± 4.09^c^
Turmeric	10.73 ± 2.28^m^	−23.79 ± 1.28^mn^
Ginger	20.04 ± 1.41^n^	−35.96 ± 1.44^ij^
Miscellaneous		
Vitamin C	−88.77 ± 0.65^a^	−88.97 ± 3.97^b^
Coffee	−42.75 ± 3.42^c^	−53.44 ± 2.85^g^
Egg yolk	−13.15 ± 1.41^hi^	−38.93 ± 6.78^i^
Coconut water	−0.23 ± 0.01^jkl^	13.95 ± 1.36^r^
Cocoa	0.75 ± 0.31^kl^	−29.04 ± 2.08^kl^
Black tea	2.87 ± 0.20^l^	5.37 ± 0.26^q^
Dates	13.55 ± 1.15^m^	−26.77 ± 2.26^lm^
Green tea	11.56 ± 1.46^m^	2.64 ± 0.36^pq^
Egg white	25.50 ± 1.41°	−3.47 ± 1.20°
Fenugreek seeds	86.79 ± 2.04^p^	−62.40 ± 2.50^ef^
Control	0.00^kl^	0.00 ± 0.00^opq^
Commercial anti-hangover product	−11.64 ± 1.49^hi^	−82.50 ± 0.57^c^

Values are mean ± SD of three determinants.

Different alphabet in superscript represent that the values are significantly different (p < 0.05).

All the cereal samples decreased ADH and ALDH activities. While maize and wheat had no significant effect on the activity of ALDH, the other samples analysed, showed a decrease in the activity of ALDH. This suggests that it is not advisable to include cereals and pulses in the diet post consumption of alcohol until the hangover has subsided.

Among the spice samples, mace, turmeric and ginger increased ADH activity. However, a commercial AHO product with turmeric as its principal component showed a decrease in the activity of ADH. The product also contained Vitamin C as one of its ingredients. The activity of ALDH was not significantly altered by cumin. Other spice samples showed a significant decrease in the activity of ALDH. The commercial AHO product was found to decrease the ALDH activity. So far, research work on effect of spices on ADH and ALDH have not been elaborately carried out and hence further studies (especially *in vivo*) need to be undertaken to establish these results.

Among the miscellaneous samples, fenugreek seeds, egg white, green tea, black tea, and dates enhanced the activity of ADH. Cocoa and coconut water did not significantly alter the activity of ADH. Ascorbic acid significantly decreased the activity of the ADH to a very large magnitude. Most of the commercial products are marketed based on their antioxidant property and these are often rich in minerals and also contain a mixture of components such as polyphenols, flavonoids and other bioactive components. In the current study, the AHO product tested did not show any positive effect on both the hepatic enzymes (ADH and ALDH). Thus, it is possible that there exists another mechanism of action for this product. A substantial decrease in the ADH and ALDH activities due to coffee indicates that consumption of coffee post alcohol consumption could slower the rate of ethanol elimination, and hence it should not be preferred during hangover. Egg white showed enhanced ADH activity, while egg yolk showed a decrease in the ADH activity. Black tea did not significantly affect the ADH activity (2.87%) while green tea showed a marginal increase in ADH activity (11.56%). These results for green tea and black tea are in agreement with Wang et al. (2016b). It was interesting to note that both green and black tea did not significantly affect the activity of ALDH. The difference in the results exhibited by green and black tea may be probably due to the difference in their polyphenols and mineral contents.

Fenugreek seeds increased the activity of ADH significantly to 86.79%. However, when fenugreek leaves were tested for their effect, no significant difference in the activities of ADH and ALDH was observed. Coconut water was found to enhance the activity of ALDH (13.95%), whereas egg white did not show any significant effect on both ADH and ALDH activities. Other samples decreased the activity of ALDH significantly. Coffee decreased the ALDH activity by a large magnitude; hence it is not advisable to consume coffee post alcohol intake as it might lead to acetaldehyde build-up resulting in prolonged hangover. It was observed that dates increased the activity of ADH but decreased the activity of ALDH. Samples that were able to enhance ADH and ALDH or only ALDH will be able to prevent acetaldehyde build-up, and thus may serve as an AHO.

### 4.3 Correlation between antioxidant property and enzyme activity

**Fig. 1:**
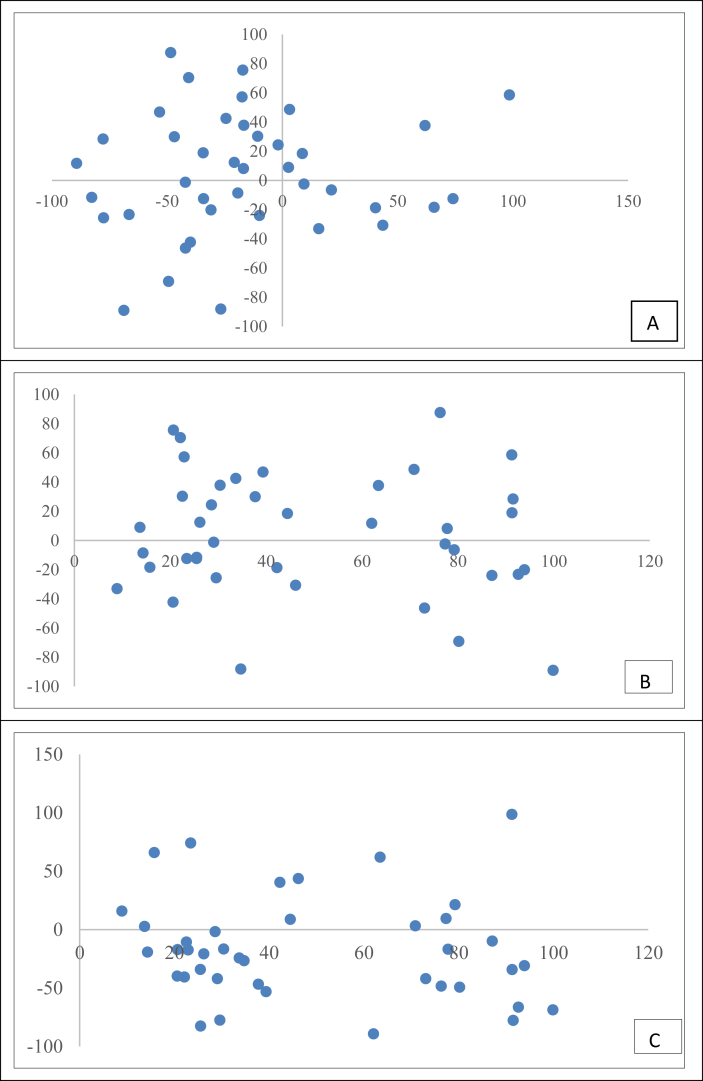
A) Correlation between alcohol dehydrogenase (ADH) and aldehyde dehydrogenase (ALDH) activity, B) radical scavenging effect and ADH activity, and C) radical scavenging effect and ALDH activity.

Selected food commodities were evaluated for antioxidant potential which is reported in supplementary file (Table S1-S6). The correlation between ADH activity, ALDH activity and percent inhibition was determined from the correlation graphs (Fig. 1.). It is evident that there was no correlation between ADH, ALDH, and antioxidant activities. The correlation between the hepatic enzymes, (ADH and ALDH) and the antioxidant property was also found to be invalid. It has been reported previously that a substance known to have high antioxidant activity can serve as an AHO as it will be able to reduce the oxidative stress induced in the body upon alcohol consumption. *Asparagus officinalis* (Kim et al., 2009) and red ginseng (Lee et al., 2014b) have been reported to have the ability to exert a positive effect on the activity of the hepatic enzymes due to their antioxidant activity. A herbal formulation, "DTS20" has been shown to prevent gastric mucous damage and also accelerate the metabolism of alcohol (Hong, 2015). Hence, it has been widely considered that compounds having antioxidant property would have the ability to enhance the activity of ADH and ALDH. However, from the results presented in Fig. 1 (A,B,C), there were hardly any correlations between the hepatic enzymes (ADH and ALDH) as well as between their activities and the antioxidant properties of the samples. These results emphasize the need to study the effect of food commodities on ADH and ALDH activities and further use them to screen for suitable AHO ingredients rather than relying solely on the antioxidant properties of substances.

### 4.4 Effect of selected food constituents on the kinetics of ADH and ALDH

**Fig. 2:**
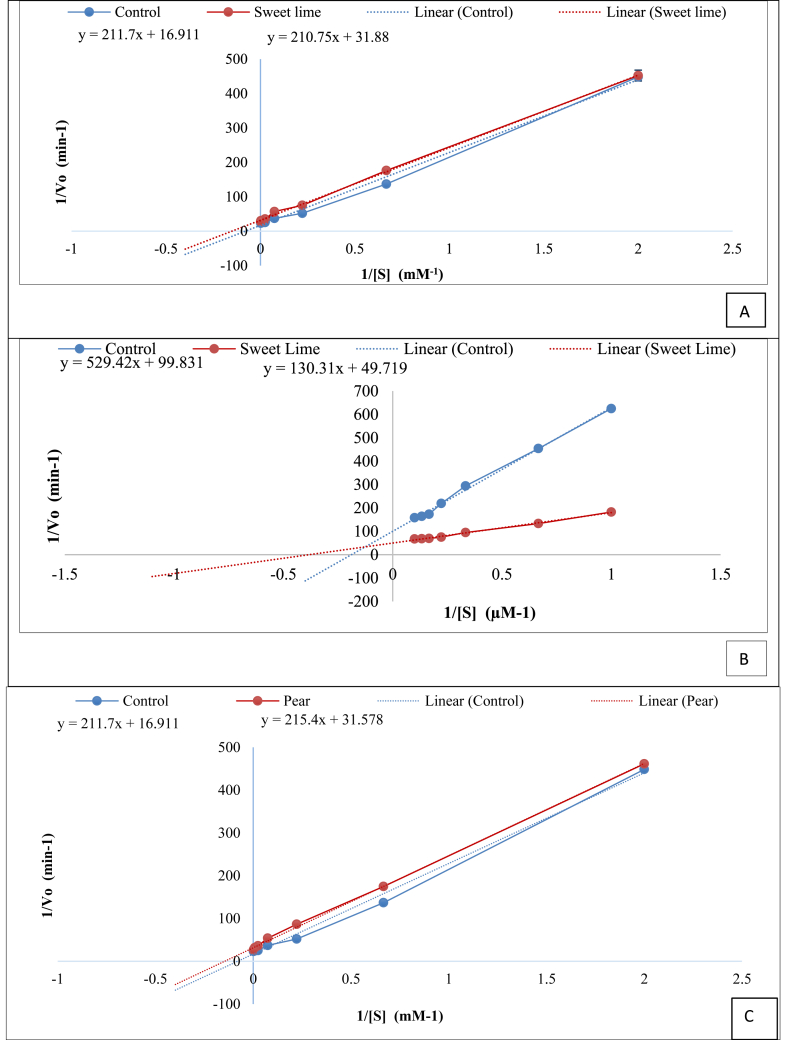

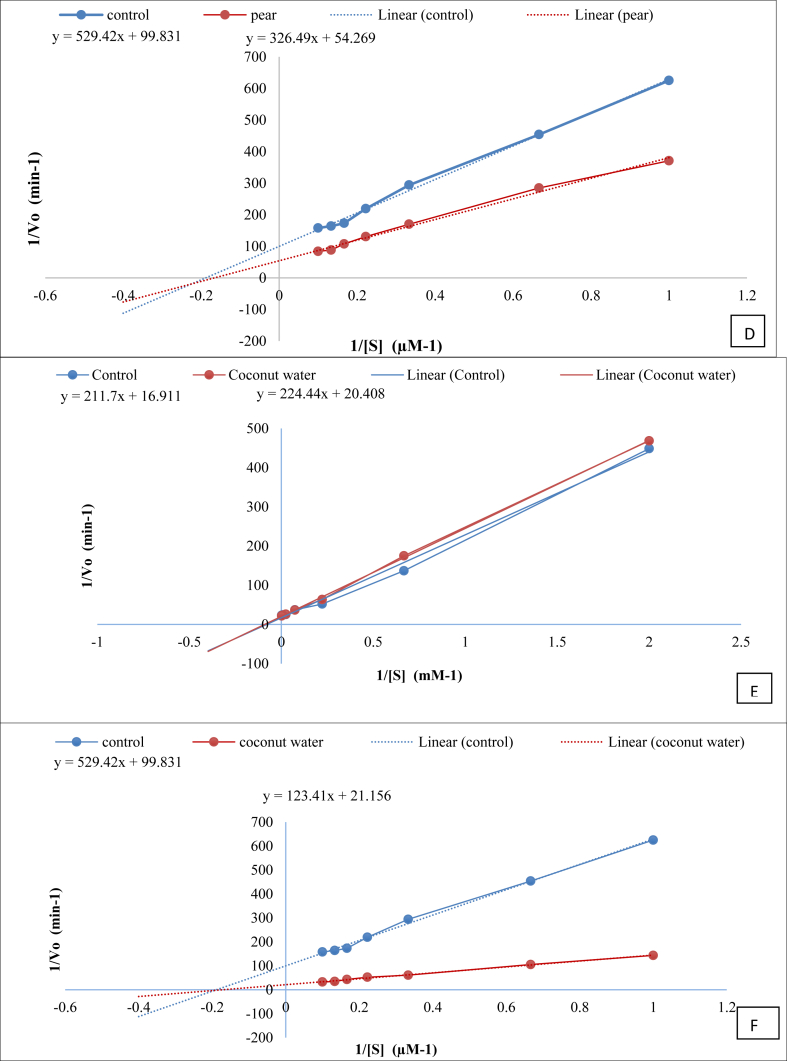
Lineweaver-Burk plot of A) aldehyde dehydrogenase (ALDH) catalyzed oxidation of ethanol in the absence (control) and presence of a modifier (sweet lime), and B) alcohol dehydrogenase (ADH) catalyzed oxidation of ethanol, in the absence (control) and presence of a modifier (sweet lime) C) aldehyde dehydrogenase (ALDH) catalyzed oxidation of ethanol in the absence (control) and presence of a modifier (pear), and D) alcohol dehydrogenase (ADH) catalyzed oxidation of ethanol, in the absence (control) and presence of a modifier (pear) E) aldehyde dehydrogenase (ALDH) catalyzed oxidation of ethanol in the absence (control) and presence of a modifier (coconut), and F) alcohol dehydrogenase (ADH) catalyzed oxidation of ethanol, in the absence (control) and presence of a modifier (coconut).

On the basis of results on enhancement of ADH and/or ALDH activities obtained in section 2.0, pear, sweet lime and coconut were selected for their effect on K_m_ of these enzymes. The presence of sweet lime reduced the K_m_ value for ADH (control) and ALDH (control) from 12.52 to 6.61 mM (Fig. 2A) and 5.30 μM to 2.62 μM (Fig. 2B), respectively. In case of pear juice, the apparent K_m_ value of ADH reduced from 12.52 mM to 6.82 mM (Fig. 2C), but did not alter the K_m_ of ALDH (Fig. 2D), while that of the control was 12.52 mM (Fig. 2C&D). Coconut water showed a very marginal decrease in K_m_ value for ADH (Fig. 2E) and almost no change in the K_m_ for ALDH activity (Fig. 2F). The decrease in the K_m_ value, correlated with the increase in ADH activity of pear and sweet lime but not with coconut water. Similarly, the increase in activity of ALDH by pear and coconut could not be explained by the kinetic parameters, suggesting that there could be other mechanisms that cause this effect.

Similarly, other food commodities were also tested for their effect on kinetic parameters (data not shown) and it was observed that the increase in enzyme activities could be correlated to kinetic parameters only for few cases suggesting other mechanisms through which the activity of the enzyme may have altered. A few studies have reported the inhibitory effect of certain food components on the kinetics of ALDH. Durian fruit has shown to have a mixed type of inhibitory action on yeast ALDH (Maninang et al., 2009), while quercetin has been shown to be a non-competitive inhibitor for ALDH (Bhuiya et al., 2017). Further studies to ascertain the role of specific molecules in the food commodities that have activated ADH and ALDH evaluated in this work need to be carried out.

### 4.5 Formulation of anti-hangover (AHO) beverage

Anti-hangover effect can be expected when there is an increase in the ADH and/or ALDH activities. Further, the rate of conversion of acetaldehyde to other metabolites must be higher than the rate of conversion of alcohol to acetaldehyde. The results obtained in this study provide an insight into the dietary components that can be used to prepare an AHO formulation.

**Fig. 3:**
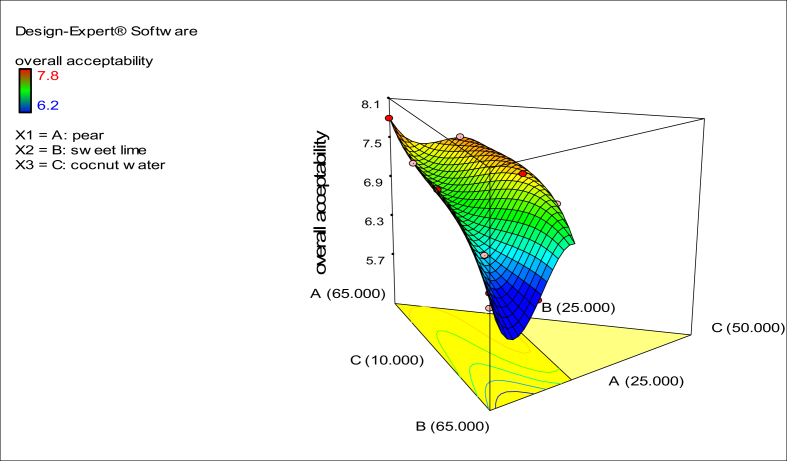
Response surface plot showing the effect of concentration of different components (pear, sweet lime, and coconut) on the overall acceptability of the anti-hangover (AHO) product.

A beverage formulation is suitable among the various food types. Hence preliminary efforts were made to incorporate fruit (sweet lime, pear, and coconut water) and vegetable (cucumber and tomato) juices in a beverage formulation. However, all formulations containing vegetable juices were not acceptable by the sensory panel. Hence three fruit juices, *viz*. sweet lime, pear, and coconut water, were selected for the AHO beverage formulation. Mango and watermelon were not selected for the beverage formulation despite their ability to enhance the activity of ADH, neither of them enhanced the activity of ALDH. This suggests that while these fruit juices can facilitate metabolism of alcohol to acetaldehyde, they would not metabolize it further to acetic acid, both of which would result in acetaldehyde build-up, and hence retention of hangover. The upper and lower limits for all three components were set according to preliminary trials (based on sensory analysis)which were set as pear (25%–65%), sweet lime (25–65%), and coconut water (10–25%). Sixteen formulations were generated with different composition of each ingredient and evaluated randomly in terms of sensory perception (Fig. 3.). The formulation containing comprising of pear (65% v/v), sweet lime (25% v/v), and coconut water (10% v/v) showed best acceptability, which was then compared with commercially available AHO (Table S7). While the formulated AHO had a higher overall acceptability (7.8), it did not differ much in terms of appearance and aroma with other formulations in this set of experiments. The formulated AHO could enhance the activity of ADH by 23.31% and that of ALDH by 70.02%, thus validating its use as an AHO product. These findings are in accordance with the other investigators who have reported effective use of Korean pear in alleviating hangover (Lee et al., 2013).

Recently, a variety of anti-hangover (AHO) products have been launched into the market. “PartySmart” is a formulation containing grapes, gooseberry, date palm, *Phyllanthus amarus*, green chiretta (*Andrographis paniculata*), and chicory, which is reported to significantly reduce several of the hangover symptoms, and prevent formation of acetaldehyde adducts. As a result, rapid elimination of acetaldehyde from the blood occurs (Ramakrishna et al., 2005). While the exact mechanism of the action by the product has not been elucidated, the study suggested the antioxidant property of the various ingredients in addition to the positive effect on ADH and ALDH by few ingredients in the product (Venkataranganna et al., 2008). Similarly, a product named “Oh!K” containing turmeric, ginger, black pepper, green tea extracts has also been formulated. Studies have shown that this drink is an effective remedy to treat alcoholic hangover by replenishing the body with the vital nutrients that are lost as a result of hangover (Gopi et al., 2014). Another product, “DotShot” containing curcumin as the principle component and other vital electrolytes, has also been reported to enhance the ALDH activity and thereby assist in the breakdown of acetaldehyde (Harisha, 2018). "LIVitup" is an AHO tablet containing *kalmegh ghan*, a mixture of *kalmegh* (*Andrographis paniculata*) and *neem* (*Azadirachta indica*) leaves which prevents the hangover symptoms by reducing the acetaldehyde build-up in the body after alcohol consumption. The fruit juice blend developed as AHO formulation in the present study is a simple, effective, economical and ready-to-prepare alternative to these formulations with good sensory appeal.

## 5 Conclusion

This study analyzed the effect of certain common food commodities on hangover through *in vitro* studies by enhancing either alcohol dehydrogenase and aldehyde dehydrogenase activities, or at least the aldehyde dehydrogenase activity, to formulate an anti-hangover product. A beverage made from a blend of sweet lime, pear, and coconut water could be used to overcome hangover. No correlation between the antioxidant activity and the activity of alcohol dehydrogenase and aldehyde dehydrogenase were seen. This dispels the common belief that an antioxidant could serve as an anti-hangover product. The consumption of this beverage with cheese, cucumber, and tomatoes may further alleviate the hangover symptoms.

## Conflict of interest

There is no conflict of interest with any individual or organization.
