# Supplementary material for: Influence of food commodities on hangover based on alcohol dehydrogenase and aldehyde dehydrogenase activities
Source: Curr Res Food Sci. 2019 Sep 17;1:8–16. doi: 10.1016/j.crfs.2019.09.001 (PMC7473379; doi:10.1016/j.crfs.2019.09.001)
Supplement: Multimedia component 1 [file mmc1.docx]

Table S1. Antioxidant activities of fruits assessed by DPPH assay

| Fruits | Antioxidant activity (mg GAE/100 mL)* |
| --- | --- |
| *Malus pumila (*Apple) | 9.28 ± 0.05 (91.57 ± 0.08) |
| *Citrullus lanatus* (watermelon) | 2.24 ± 0.08 (21.96 ± 0.15) |
| *Mangifera indica* (mango) | 3.83 ± 0.09 (37.49 ± 0.04) |
| *Carica papaya* (papaya) | 3.99 ± 0.06 (39.33 ± 0.14) |
| *Vitis vinifera* (green grapes) | 8.47 ± 0.01 (83.53 ± 0.10) |
| *Phyllanthus emblica* (gooseberry) | 5.12 ± 0.12 (50.53 ± 1.17) |
| *Ananas comosus* (pineapple) | 3.42 ± 0.05 (33.69 ± 0.52) |
| *Punica granatum* (pomegranate) | 2.10 ± 0.02 (20.67 ± 0.05) |
| *Citrus limon* (L.) (lemon) | 2.32 ± 0.08 (22.08 ± 0.05) |
| *Actinidia deliciosa* (kiwifruit) | 3.07 ± 0.01 (30.28 ± 0.14) |
| *Vitis amurensis* (black grapes) | 7.85 ± 0.03 (77.43 ± 0.26) |
| *Hylocereus undatus* (pitahaya/dragonfruit) | 2.95 ± 0.05 (29.08 ± 0.50) |
| *Citrus sinensis* (L.) (orange) | 8.03 ± 0.00 (79.24 ± 0.02) |
| *Averrhoa carambola* (carambola/starfruit) | 3.94 ± 0.22 (38.85 ± 2.13) |
| *Citrus limetta* (sweet lime) | 6.43 ± 0.08 (63.72 ± 0.04) |
| *Pyrus sp.* (pear) | 4.51 ± 0.01 (44.46 ± 0.12) |

Values are mean ± SD of three determinants

*Values within parenthesis are given as percent inhibition

Table S2. Antioxidant activities of vegetables assessed by DPPH assay

| Vegetables | Antioxidant activity (mg GAE/100 mL)* |
| --- | --- |
| *Coriandrum sativum* (coriander) | 4.04 ± 0.09 (39.83 ± 0.91) |
| *Spinacia oleracea* (spinach) | 3.53 ± 0.08 (34.79 ± 0.79) |
| *Allium sativum* (garlic) | 2.09 ± 0.04 (20.61 ± 0.42) |
| *Momordica charantia* (bitter gourd/bitter melon) | 0.24 ± 0.00 (2.39 ± 0.04) |
| *Trigonella foenum-graecum* (fenugreek) | 8.29 ± 0.02 (81.81 ± 0.15) |
| *Carrot* | 2.29 ± 0.04 (22.08 ± 0.05) |
| *Allium cepa* (Onion) | 7.19 ± 0.05 (70.87 ± 0.47) |
| *Solanum lycopersicum* (tomato) | 9.25 ± 0.06 (91.24 ± 0.64) |
| *Cucumis sativus* (cucumber) | 1.62 ± 0.01 (15.96 ± 0.05) |

Values are mean ± SD of three determinants

*Values within parenthesis are given as percent inhibition

Table S3 Antioxidant activities of cereals and pulses assessed by DPPH assay

| Cereals and pulses | Antioxidant activity (mg GAE/100 g)* |
| --- | --- |
| Bajra | 88.41 ± 1.69 (29.65 ± 0.17) |
| Jowar | 140.42 ± 0.76 (46.00 ± 0.10) |
| Maize | 71.32 ± 1.58 (23.45 ± 0.52) |
| Oats | 90.03 ± 1.87 (16.57 ± 0.32) |
| Peanut | 104.23 ± 0.61 (34.27 ± 0.20) |
| Wheat | 128.56 ± 2.78 (41.29 ± 0.05) |

Values are mean ± SD of three determinants.

*Values within parenthesis are given as percent inhibition

Table S4 Antioxidant activities of dairy products assessed by DPPH assay

| Dairy products | Antioxidant activity (mg GAE/100 mL)* |
| --- | --- |
| Buttermilk | 0.90 ± 0.06 (9.02 ± 0.06) |
| Cheese | 4.17 ± 0.12 (4.56 ± 0.08) |
| Milk | 1.45 ± 0.09 (14.51 ± 0.16) |
| Yakult | 31.36 ± 0.08 (77.53 ± 0.09) |

Values are mean ± SD of three determinants.

*Values within parenthesis are given as percent inhibition

Table S5 Antioxidant activities of spices assessed by DPPH assay

| Spices | Antioxidant activity  (mg GAEC/100 g)* |
| --- | --- |
| Ginger | 9.26 ± 0.01^**^ (91.29 ± 0.13) |
| Cinnamon | 187.77 ± 1.67 (91.72 ± 0.06) |
| Cloves | 163.82 ± 2.21 (80.16 ± 0.05) |
| Mace | 126.89 ± 1.55 (62.12 ± 0.11) |
| Cassia | 189.56 ± 1.83 (94.04 ± 0.13) |
| Cumin | 176.61 ± 1.23 (87.61 ± 0.06) |
| Turmeric | 157.71 ± 1.32 (77.61 ± 0.15) |
| Black pepper | 70.38 ± 1.08 (34.08 ± 0.04) |
| Nutmeg | 188.07 ± 0.20 (92.75 ± 0.10) |

Values are mean ± SD of three determinants

*Values within parenthesis are given as percent inhibition

**Value represented as mgGAE/100 ML

Table S6 Antioxidant activities of few miscellaneous products assessed by DPPH assay

| Food commodities | Antioxidant activity (mg GAE/100 g)* |
| --- | --- |
| Ascorbic acid | 50633.72 ± 7.64 (99.89 ± 0.01) |
| Cocoa | 79.25 ± 2.29 (45.23 ± 0.63) |
| Coconut water | 3.67 ± 0.07^**^ (36.22 ± 0.68) |
| Coffee | 987.08 ± 0.54 (73.02 ± 0.04) |
| Egg white | 2.90 ± 0.05^**^ (28.98 ± 0.21) |
| Egg Yolk | 25.85 ± 1.20 (26.86 ± 0.15) |
| Tea | 434.60 ± 11.08 (32.15 ± 0.82) |
| Dates | 26.57 ± 1.05 (27.18 ± 0.10) |
| Fenugreek seeds | 154.73 ± 2.40 (76.31 ± 1.18) |
| Green tea | 976.40 ± 12.57 (72.23 ± 0.93) |
| Commercial anti-hangover product | 10.35 ± 0.04^**^ (25.53 ± 0.11) |

Values are mean ± SD of three determinants.

*Values within parenthesis are given as percent inhibition,

**Value represented as mgGAE/100 mL

**Table S7**

Mean sensory scores of formulated anti-hangover product and a commercial anti-hangover product

| Product | Appearance | Aroma | ^*^Taste | ^**^Mouthfeel | ^***^Overall acceptability |
| --- | --- | --- | --- | --- | --- |
| AHO | 6.3 ± 0.82 | 6.1 ± 0.74 | 7.4 ± 0.84 | 7.3 ± 0.95 | 7.7 ± 0.67 |
| Commercial | 6.6 ± 0.52 | 5.9 ± 0.88 | 6.0 ± 1.05 | 5.8 ± 0.79 | - 1. ± 0.48 |

Values are presented as mean ± SD determined for fifteen panellists

^*,**,***^Values in column differ significantly (p<0.05)
